# Intricate Evolution of Multifunctional Lipoxygenase in Red Algae

**DOI:** 10.3390/ijms252010956

**Published:** 2024-10-11

**Authors:** Zhujun Zhu, Yanrong Li, Xinru Wu, Jia Li, Xiaodong Mo, Xiaojun Yan, Haimin Chen

**Affiliations:** 1 Marine Drugs and Biological Products Department, Ningbo Institute of Oceanography, Ningbo 315832, China; zhuzj@nbio.org.cn (Z.Z.); liyr@nbio.org.cn (Y.L.); 2 State Key Laboratory for Managing Biotic and Chemical Threats to the Quality and Safety of Agro-Products, Ningbo University, Ningbo 315832, China; 13216665758@163.com (X.W.); azurelone@hotmail.com (J.L.); moxiaodong1221@163.com (X.M.); 3 Collaborative Innovation Center for Zhejiang Marine High-Efficiency and Healthy Aquaculture, Ningbo University, Ningbo 315832, China; yanxiaojun@nbu.edu.cn

**Keywords:** fatty acid oxidation, cytochrome P450, lipoxygenase, multifunctional enzyme, protein evolution, algae

## Abstract

Lipoxygenases (LOXs) from lower organisms have substrate flexibility and function versatility in fatty acid oxidation, but it is not clear how these LOXs acquired the ability to execute multiple functions within only one catalytic domain. This work studied a multifunctional LOX from red alga *Pyropia haitanensis* (PhLOX) which combined hydroperoxidelyase (HPL) and allene oxide synthase (AOS) activity in its active pocket. Molecular docking and site-directed mutagenesis revealed that Phe642 and Phe826 jointly regulated the double peroxidation of fatty acid, Gln777 and Asn575 were essential to the AOS function, and the HPL activity was improved when Asn575, Gln777, or Phe826 was replaced by leucine. Phylogenetic analysis indicated that Asn575 and Phe826 were unique amino acid sites in the separated clades clustered with PhLOX, whereas Phe642 and Gln777 were conserved in plant or animal LOXs. The N-terminal START/RHO_alpha_C/PITP/Bet_v1/CoxG/CalC (SRPBCC) domain of PhLOX was another key variable, as the absence of this domain disrupted the versatility of PhLOX. Moreover, the functions of two homologous LOXs from marine bacterium *Shewanella violacea* and red alga *Chondrus crispus* were examined. The HPL activity of PhLOX appeared to be inherited from a common ancestor, and the AOS function was likely acquired through mutations in some key residues in the active pocket. Taken together, our results suggested that some LOXs from red algae attained their versatility by amalgamating functional domains of ancestral origin and unique amino acid mutations.

## 1. Introduction

Lipoxygenases (LOXs), which serve as pivotal enzymes in the oxidative metabolism of polyunsaturated fatty acids (PUFAs), are prevalent in prokaryotes, plants, and animals. They act in coordination with specific cytochrome P450 proteins, notably CYP74, to synthesize various oxylipins and play crucial roles in defense responses against biotic and abiotic stress [1,2]. In plants, allene oxide synthase (AOS), hydroperoxide lyase (HPL), and divinyl ether synthase (DES) are all key CYP74 enzymes of oxylipin metabolism involved in the biosynthesis of jasmonic acid (JA), green leaf volatiles (GLVs), and divinyl ether fatty acids [3]. Marine algae live in dynamic seawater environments and use oxylipin mechanisms to cope with environmental stress [4,5,6]. Metabolic studies on red and brown algae have revealed a rich array of hydroperoxides, prostaglandins, short-chain aldehydes, and alcohols [7], but there is scant information regarding the enzymes responsible for the biosynthesis of these interesting compounds. Genomic investigations of some representative red algae, including Bangiophyceae *Porphyra umbilicalis*, *Pyropia yezoensis*, and *P. haitanensis*, as well as Florideophyceae *Chondrus crispus*, have identified only a limited number of genes encoding LOX [8]. Notably, no candidates for AOS, HPL, and DES have been reported in these red algae [9], making the synthesis of these oxylipins somewhat difficult to understand.

In 2015, our group identified from red algae *P. haitanensis* two lipoxygenases possessing remarkable multifunctionality [10,11]. These enzymes, referred to as PhLOX and PhLOX2, are encoded by two genes transferred horizontally from marine bacteria [8], and one of them, i.e., PhLOX, combines unusually high HPL, LOX, and AOS activity within only one single catalytic domain. Additionally, both of them exhibit flexibility in substrate specificity and oxygenation positions, in sharp contrast to the highly specific catalytic functions and positions observed in the LOXs from higher animals and plants. These findings could to some extent account for why red algae lack HPL and AOS enzymes but still produce diverse oxylipins. Therefore, investigating the biological origin and evolution of the versatility of LOXs in red algae is a compelling topic of interest.

Some other lower organisms that have fusion genes or a single gene for a bifunctional enzyme also generate LOXs with multifunctional properties [12]. For example, a LOX from the alga *Chlorella pyrenoidosa* shows HPL activity under oxygen deprivation [13]. HPL activity also exists in PpLOX1 from the moss *Physcomitrella patens* [14]. The catalase-like AOS-LOX fusion protein from cyanobacteria and corals, which comprises a monomeric LOX fused with a heme-containing AOS, is encoded by two separate genes that form an operon [15,16]. These findings suggest that in the early stages of biological evolution, LOXs may have possessed functional diversity, and that it was only during the later stages of evolution that functional differentiation emerged between plant and animal LOXs.

It has been argued that LOX, AOS, and HPL enzymes in the oxylipin pathway probably share a common primitive ancestor, and over the long course of evolution, the divergence of gene coding sequences has led to some fundamental changes resulting in the differentiation of enzyme function [17]. AOS and HPL are heme proteins [18], but LOXs are nonheme iron-containing dioxygenases [19]. Therefore, in lower organisms, multifunctional LOXs holding one nonheme iron in the active pocket have no relationship with the actual hemoproteins of the CYP74 family even if they catalyze the equivalent reactions of AOS or HPL. Several amino acid residues in the LOXs from animals and higher plants are believed to determine the stereospecificity and regiospecificity of PUFA oxidation derivatives [20,21,22,23,24]. For example, in rabbit reticulocyte 15-LOX, Phe353, Glu357, Arg403, Ala404, Ile418, and Ile59 are jointly involved in substrate orientation when arachidonic acid (ARA) enters the active pocket [22]. However, these results only address substrate binding in the active pocket and the specificity of fatty acid oxidation mediated by LOX, and they do not shed light on the versatility of LOX in lower organisms. While lower organisms commonly adapt to complex environments with a very compact genome, enzymes like PhLOX have not been reported. The unprecedented multifunctionality of PhLOX may have evolved from genetic mutation at certain key sites in a common ancestor or have been inherited from an ancient but special ancestor.

In this work, the key variables affecting the functions of PhLOX were determined by molecular docking and mutagenic studies. The enzymatic functions of the LOX genes from the marine bacterium *Shewanella violacea* and the red alga *C. crispus*, both of which are clustered in an independent clade with PhLOX, were identified to study the evolutionary relationship of multifunctional LOX from a common ancestor. The results help to clarify the relationship between the evolution of the oxylipin pathway and environmental adaptation in algae.

## 2. Results

### 2.1. Computational Prediction of the Potential Positional Determinants in PhLOX

**Figure 1:**
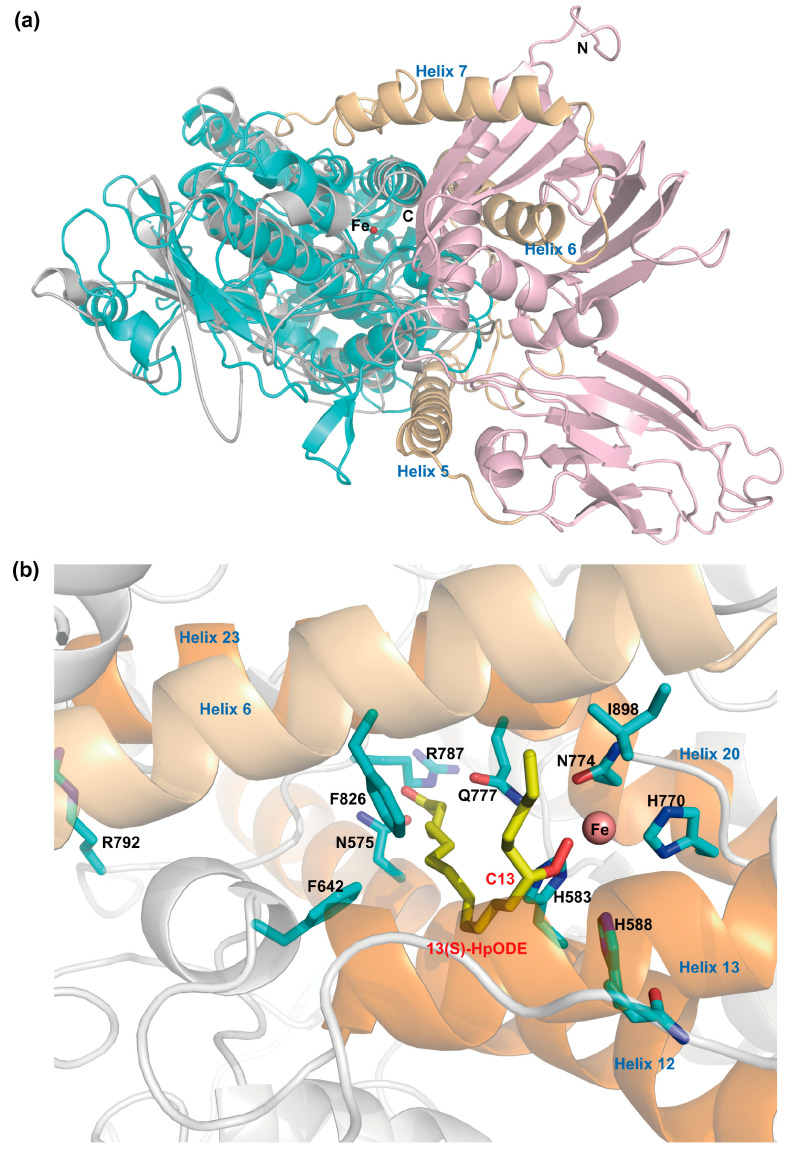
Structural modeling of PhLOX. (**a**) The 3D model constructed by Robetta is colored in light pink, tan, and cyan from the N-terminal to the C-terminal. The homology model based on rabbit 15-LOX (PDB: 1LOX) is drawn in gray. The Fe atom is the red sphere in both models. (**b**) The 13(S)-hydroperoxy-9(Z),11(E)-octadecadienoic acid ligand (i.e., 13(S)-HpODE) extracted from soybean LOX3 (PDB: 1IK3) and the enzyme side chains in the 3D model constructed by Robetta. The Fe metal center is paired with active site residues, including His583, His588, His770, Asn774, and Ile898 (i.e., H583, H588, H770, N774, and I898). The side chains of Phe642 (F642) and Phe826 (F826) prevent the ligand from adopting a linear orientation and reaching Arg792 (R792) at the bottom of the active pocket. The carboxyl end of the ligand is surrounded by Asn575 (N575) and Arg787 (R787). The side chain of Gln777 (Q777), a residue in helix 20, points to the hydroperoxyl group in 13(S)-HpODE.

A 3D model of PhLOX is constructed after the ab initio algorithm in Robetta and used to predict the tertiary protein structure (Figure 1a). In the 3D model, the N-terminal portion (1–284 aa) forms two perpendicular beta barrels and is connected to the LOX domain at the C-terminal portion (424–898 aa) through three alpha helices (helix 5–7). The N-terminal portion is a START/RHO_alpha_C/PITP/Bet_v1/CoxG/CalC (SRPBCC) domain (Figure S1). The LOX domain primarily consists of 18 alpha helices and harbors an active pocket surrounded by helix 12, 13, 20, and 23 (Figure 1b), and the entrance of the pocket is very close to helix 6. When the 3D model of PhLOX is aligned to soybean LOX-3 (PDB: 1IK3, 30.1% similarity), the Fe metal extracted from soybean LOX-3 is well coordinated with His583, His588, His770, Asn774, and Ile898 in the active pocket of PhLOX. In addition, the carboxyl end of the 13(S)-hydroperoxy-9(Z),11(E)-octadecadienoic acid ligand (i.e., 13(S)-HpODE) is surrounded by Asn575 and Arg787, and the side chain of Gln777 points to the oxygen of the hydroperoxyl group at C13. The bulky side chains of Phe642 and Phe826 are located between 13(S)-HpODE and Arg792 as a “Sloane determinant” [25]. A homologous search in the PDB database shows that the LOX domain of PhLOX shares the highest sequence homology with rabbit 15-LOX (PDB: 1LOX, 30.8%). The homology model based on rabbit 15-LOX has high similarity to the 3D model of PhLOX constructed by Robetta (Figure 1a).

### 2.2. Mutagenesis of Phe642 and Phe826 in PhLOX Disrupts the Double Peroxidation

**Figure 2:**
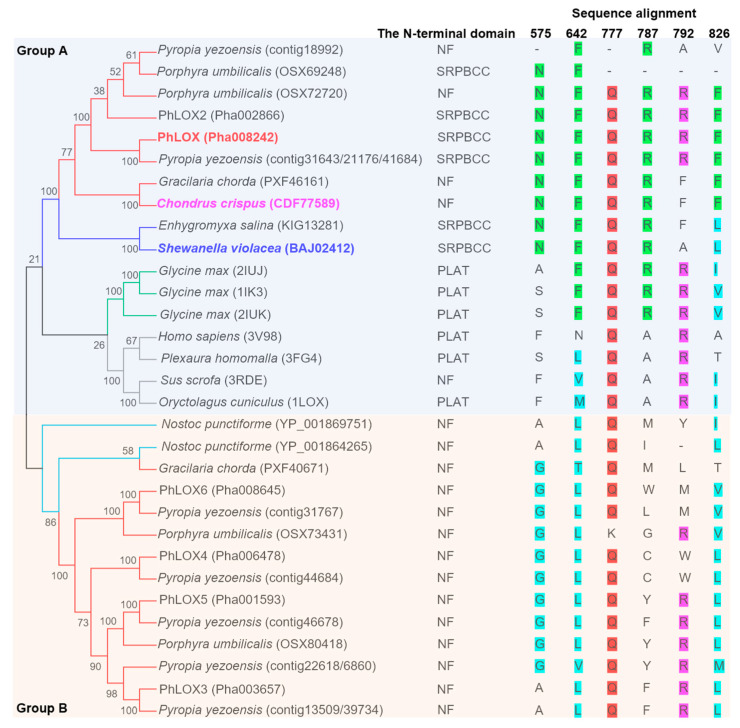
Phylogenetic analyses and multiple sequence alignment of the LOX genes from different species. Red branches are red algal genes, green branches are genes from higher plants, blue branches are bacterial genes, cyan branches are genes from *Nostoc punctiforme*, and gray branches are genes from animals. PhLOX and the homologs from *Chondrus crispus* and *Shewanella violaea* are typeset in bold and colored in red, pink, and blue, respectively. At the top row are the numbers corresponding to the amino acid sites in PhLOX, under which letters are marked with green, cyan, red, and pink to denote the conserved sites in different species. The N-terminal domain is annotated as “conserved domains” in NCBI for all LOX protein sequences.

In the multiple sequence alignment analyses of LOX genes, the Phe aligned to the 826th site of PhLOX appears to be strictly conserved in red algae LOX genes clustered in Group A, and another Phe aligned to the 642th site of PhLOX is present in all Group A genes except those from animals (Figure 2). In addition, the Arg at the bottom of the active pocket (i.e., Arg792 in PhLOX) appears more divergent in the LOX genes from Prokaryotes and Rhodophytes than those from higher plants and animals.

**Figure 3:**
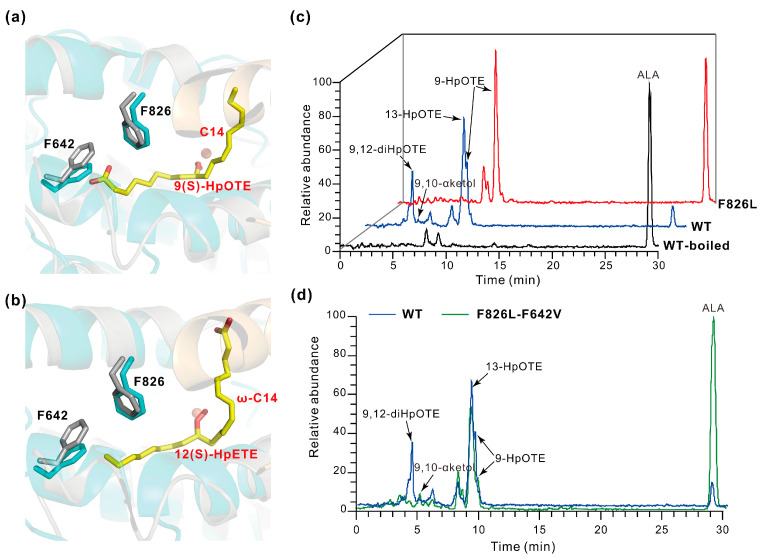
Molecular docking of the intermediates in dihydroperoxide formation and HPLC-MS detection of α-linolenic acid (ALA) oxidation products. (**a**,**b**) The docking of 9(S)-hydroperoxy-10(E),12(Z),15(Z)-octadecatrienoic acid and 12(S)-hydroperoxy-5(Z),8(Z),10(E),14(Z)-eicosatetraenoic acid (i.e., 9(S)-HpOTE and 12(S)-HpETE) in the active pocket. The ligands and the side chains of F642 and F826 are shown as sticks. The benzene ring of F826 points to the C14 of 9(S)-HpOTE and the ω-C14 of 12(S)-HpETE. The benzene ring of F642 prevents the substrate from adopting a linear orientation within the active pocket. (**c**,**d**) Oxidation of ALA catalyzed by the F826L mutant or the F642V-F826L double mutant. ALA (100 μM) was incubated with the enzyme (0.1 mg·mL^−1^) at pH 8.0 and 20 °C for 15 min. Detection was made for *m*/*z* = 270–380.

The double peroxidation of some octadecatrienoic and eicosatetraenoic acids mediated by LOXs may have hydroperoxyl derivatives as the intermediate [10,23]. Here, the formation pattern of 9,12-dihydroperoxide was examined using 9(S)-hydroperoxy-10(E),12(Z),15(Z)-octadecatrienoic acid (i.e., 9(S)-HpOTE) as substrate of PhLOX in vitro, and a high rise in 9,12-dihydroperoxy octadecatrienoic acid (9,12-diHpOTE) and a little production of 9,12-αketol were detected in the HPLC spectra (Figure S2), consistent with these previous reports. When the carboxyl end of 9(S)-HpOTE or the methyl end of 12(S)-hydroperoxy-5(Z),8(Z),10(E),14(Z)-eicosatetraenoic acid (i.e., 12(S)-HpETE) enters the active pocket by docking, the benzene ring of Phe826 pointing to the C14 of 9(S)-HpOTE or the ω-C14 of 12(S)-HpETE forces the molecule into a bent orientation (Figure 3a,b). When α-linolenic acid (ALA) was incubated with the F826L mutant of PhLOX, much more 9-HpOTE was formed but the double peroxidation product 9,12-diHpOTE could not be detected (Figure 3c, Table S1). That is, the mutant lost its ability to give the dihydroperoxide product after leucine replaced Phe826. When ALA was incubated with the F642V-F826L double mutant of PhLOX, 13-HpOTE was the dominating product, and the dihydroperoxide products were not detected (Figure 3d). Presumably, replacing Phe642 and Phe826 with smaller amino acids exposes the charged arginine residue Arg792, which allows the carboxylic group of the substrate to insert deeper and facilitates oxygen insertion at C13.

### 2.3. Asn575 and Gln777 Jointly Regulate the AOS Function of PhLOX

The AOS function of PhLOX can be reflected by the small amounts of α- and γ-ketols in the oxidation products, as they are nonenzymatic derivatives of allene oxides [10]. Under the catalytic action of PhLOX (the wild type), the relative abundance of all ketol peaks was increased significantly (Figure S3), compared with the control groups with inactive PhLOX as catalyst. Furthermore, an isotopically labeled ketol, 9,12-γketol-[D8] was also detected when isotopically-labeled [D8]-arachidonic acid (ARA-[D8]) was used as substrate for PhLOX, suggesting that the formation of ketol products could be mediated by the AOS activity of PhLOX.

**Figure 4:**
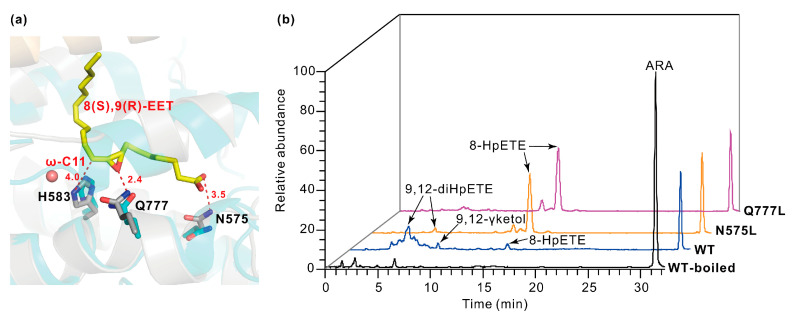
Molecular docking of the intermediates in ketol formation and HPLC-MS detection of arachidonic acid (ARA) oxidation products. (**a**) The docking of 8(S),9(R)-epoxy-5(Z),11(Z),14(Z)-eicosatrienoic acid (i.e., 8(S),9(R)-EET) in the active pocket. 8(S),9(R)-EET is an allene oxide derivative of 8-HpETE, an 8-hydroperoxy-C20:4 fatty acid. The ligand and the side chains of N575, H583, and Q777 are shown as sticks. The side chain of Q777 points to the oxygen of the epoxide from ~2.4 Å away. The imidazole ring of H583 points to the ω-C11 of 8(S),9(R)-EET from ~4.0 Å away. The amide nitrogen of N575 is ~3.5 Å away from the carboxylic group of 8(S),9(R)-EET. (**b**) Oxidation of ARA catalyzed by the N575L or the Q777L mutant. ARA (100 μM) was incubated with the enzyme (0.1 mg·mL^−1^) at pH 8.0 and 20 °C for 1 min. Detection was made for *m*/*z* = 270–380.

8(S),9(R)-epoxy-5(Z),11(Z),14(Z)-eicosatrienoic acid (i.e., 8(S),9(R)-EET) is an allene oxide derivative of 8-HpETE, an 8-hydroperoxy-C20:4 fatty acid. When inserted suitably into the active pocket, 8(S),9(R)-EET adopts a bent geometry by folding at ω-C11, which is about ~4.0 Å from the imidazole ring of His583 (Figure 4a). The side chain of Gln777 is ~2.4 Å away from the oxygen of the epoxide, and it is likely involved in the AOS function of PhLOX. Indeed, the AOS activity was not observed when ARA was incubated with the Q777L mutant of PhLOX, as no ketol was formed and 8-HpETE was the dominant product (Figure 4b, Table S1). However, the AOS function cannot be attributed to Gln777 alone, as animal and plant LOX also exhibit the conservation of glutamine at the 777th site but only PhLOX has an AOS function (Figure 2). In fact, the AOS function may be related to substrate orientation, since the carboxylic group of 8(S),9(R)-EET is ~3.5 Å to the nitrogen on the amide group of Asn575 (Figure 4a), and Asn575 is a unique amino acid in the LOX of red algae and two marine bacteria clustered in Group A (Figure 2). Indeed, when ARA was incubated with the N575L mutant of PhLOX, there was a reduction in the ketol peak and a remarkable increase in the 8-HpETE peak in LC/MS (Figure 4b), suggesting that the AOS activity dropped significantly when Asn575 was exchanged for a neutral residue like leucine. Presumably, Asn575 is a positioning factor that affects the substrate orientation to regulate the AOS function.

### 2.4. Replacement of Asn575, Gln777, or Phe826 with a Neutral Residue and Deletion of the SRPBCC Domain Change the HPL Activity of PhLOX

**Figure 5:**
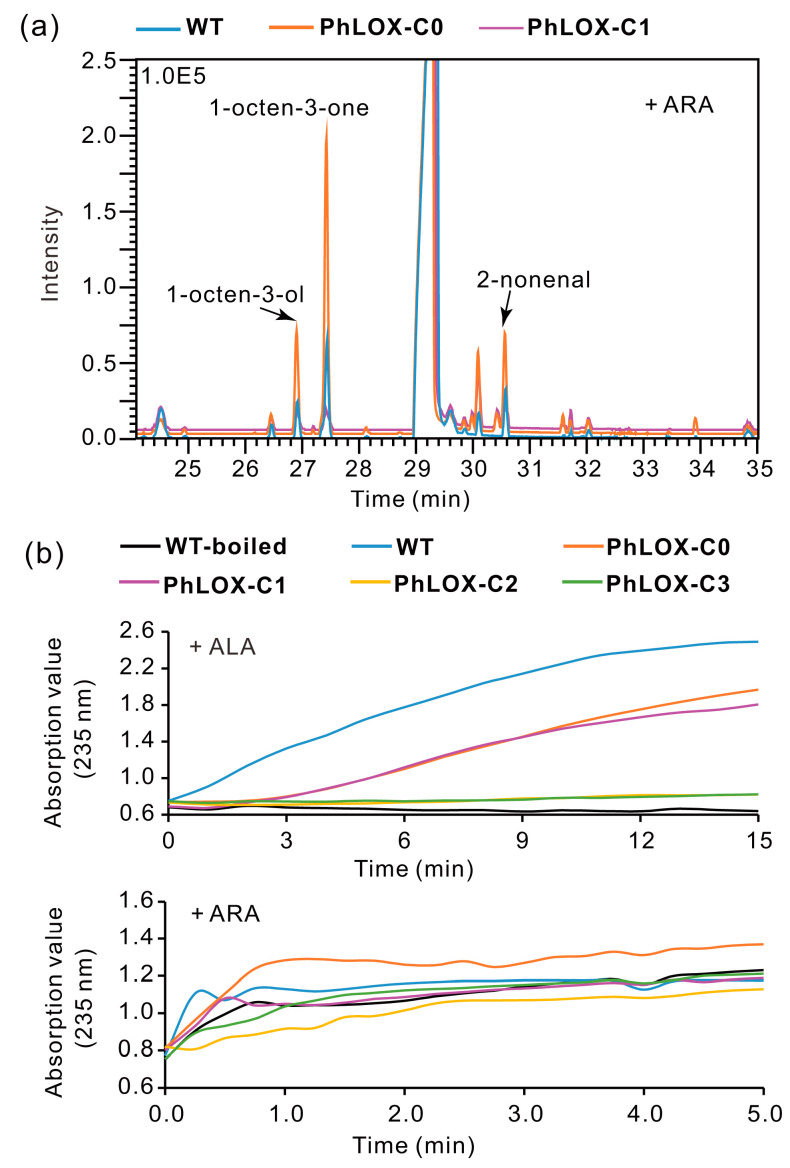
VOC production and enzyme activity of PhLOX of different size. (**a**) The volatile products from ARA using PhLOX-C0 and PhLOX-C1. ARA (100 μM) was incubated with the enzyme (0.1 mg·mL^−1^) at pH 8.0 and 20 °C for 15 min. (**b**) The activity array for PhLOX isoforms of different size without the N-terminal SRPBCC domain. ALA or ARA (100 μM) was incubated with the enzyme (0.1 mg·mL^−1^) at pH 8.0 and 20 °C. The control group used boiled wild-type PhLOX. UV absorption was measured at 235 nm, which is the absorption wavelength for hydro(pero)xidized fatty acids.

C8-VOCs were produced when 12(S)-HpETE was used as substrate of PhLOX (Figure S4), indicating that PhLOX incorporated HPL activity into its LOX active pocket. GC-MS also revealed that more volatiles were formed when mutation occurred in Asn575, Gln777, or Phe826 (Table 1, Figure S5), suggesting that the removal of these amino acid residues strengthened the HPL activity of PhLOX. In addition, when ARA was incubated with PhLOX-C0, in which the first beta barrel in the SRPBCC domain was deleted (Figure S6), more volatile products were detected by GC-MS (Figure 5a), which implies stronger HPL function. However, no VOCs were detected if both Phe642 and Phe826 were replaced by leucine (i.e., F642V-F826L) and if the whole SRPBCC domain was deleted (i.e., PhLOX-C1), which implies the loss of HPL activity.

Interestingly, by measuring the UV absorption of hydroperoxyl products at 235 nm, the absorption curves of PhLOX-C0 and PhLOX-C1 showed a sharp decrease in the maximum absorption value (with ALA as substrate) or a significant delay in the absorption peak (with ARA as substrate) compared with the wild type (Figure 5b). This suggested that their fatty acid peroxidation activity was only partially destroyed. However, the enzymes that had the connecting portion of the alpha helices removed (PhLOX-C2 and PhLOX-C3) lost their essential fatty acid peroxidation activity because their absorption curves are barely elevated. That is, the connecting alpha helices must be critical for maintaining the correct spatial conformation of the active pocket in PhLOX.

In fact, according to the data of *P. haitanensis* in the PacBio Iso-Seq Library (BioProject accession no. PRJNA717322) reported by Chen et al. [8], the *Phlox* gene produces a total of nine transcript isoforms during heteromorphic alternation of generation (Figure S7). Among them, three isoforms (Pha008242, S-5.352.3, and S-5.352.4) can give protein products containing double domains (the wild type), while the mRNA of two other isoforms (S-5.352.6 and S-5.352.8) only encodes proteins with the C-terminal lipoxygenase domain (the C-type). Hence, the N-terminal SRPBCC domain of PhLOX may be involved in the regulation of oxylipin metabolism in *P. hatanensis*.

### 2.5. The HPL Function of PhLOX Is Inherited from the Common Ancestor of Marine Bacteria LOX

**Figure 6:**
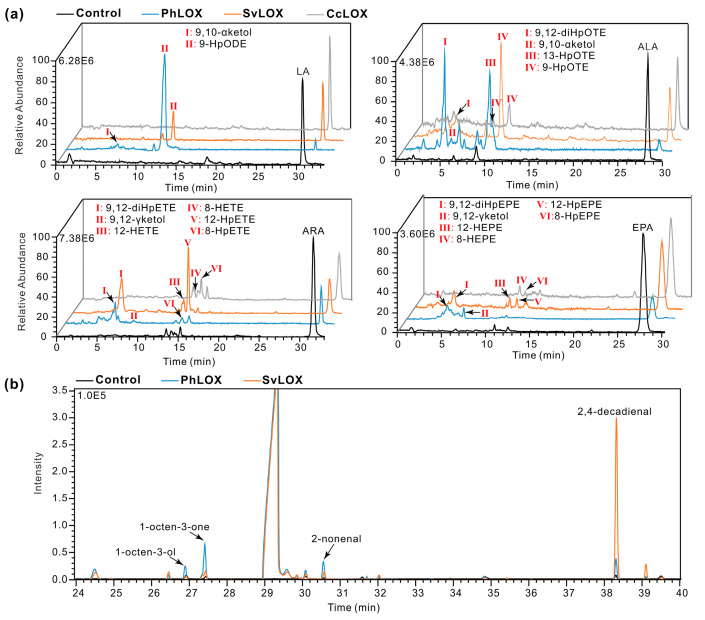
Incubation of fatty acids with SvLOX and CcLOX. (**a**) Incubation of different fatty acids (100 μM) with PhLOX, SvLOX, and CcLOX (0.1 mg·mL^−1^) at pH 8.0 and 20 °C. The incubation time was 15 min for C18 fatty acids and 1 min for C20 fatty acids. (**b**) GC-MS detection of the volatile products after ARA (100 μM) was incubated with PhLOX or SvLOX (0.1 mg·mL^−1^) at pH 8.0 and 20 °C for 15 min.

The SRPBCC domain is present in the LOX genes from the independent clade clustered within two marine bacteria and Bangiophyceae but not in the LOX genes of other red algae (Figure 2). To investigate the multifunctional evolution of PhLOX, we selected and identified *S. violaea* LOX (SvLOX) and *C. crispus* LOX (CcLOX) to test their enzymatic activity on fatty acid peroxidation. SvLOX resembled PhLOX with respect to substrate flexibility and product diversity (Figure 6a, Table S1). For example, the incubation of ARA with SvLOX gave 12-hydroperoxy/hydroxy eicosatetraenoic acids (12-HpETE/12-HETE) and 9,12-hydroperoxy eicosatetraenoic acid (9,12-diHpETE), which represented the production of hydroperoxides, hydrogenated derivatives, and double hydroperoxides from the C20 fatty acid, respectively. Therefore, SvLOX had both LOX and hydroperoxidase activity. Interestingly, the accumulation of 2,4-decadienal was observed when ARA was incubated with SvLOX (Figure 6b), indicating that SvLOX also had HPL activity. However, when ALA, ARA, and eicosapentaenoic acid (EPA) were incubated with CcLOX, only a small amount of 9-HpOTE, 8-HpETE/8-HETE, and 8-HpEPE/8-HEPE (a 8-hydroperoxy/hydroxy-C20:5 fatty acid) was detected, respectively (Figure 6a, Table S1), and there were neither new volatile products nor ketol. Therefore, CcLOX had neither AOS nor HPL functionality.

## 3. Discussion

Four amino acid residues (i.e., Asn575, Phe642, Gln777, and Phe826) have been identified by modeling to be involved in substrate orientation. Among them, Gln777 is conserved among different evolutionary clades, Asn575 and Phe826 only appear in separated clades clustered with PhLOX, and Phe642 appears in clades clustered with PhLOX or higher plants. Molecular docking suggests that Phe826 determines the substrate orientation to position the allylic methylene C14 (in C18:3 fatty acid) or ω-C14 (in C20:4 fatty acid) close to the nonheme iron, to facilitate the formation of the corresponding 9,12-dihydroperoxide. The bulky side chain of Phe642 forces the substrate to adopt a bent orientation within the active pocket of PhLOX, which prevents the interaction between Arg792 and the carboxylic end of the fatty acids. The above analyses were confirmed by site-directed mutagenesis studies. The “flip” model has been used to explain the production of dihydroperoxide products, and in the case of soybean LOX-1, the initial oxygenation of ARA to form 15(S)-HpETE is followed by the reversal of its orientation (i.e., the “flip”), and the 5S or 8S oxygenation will then give 5(S),15(S)-diHpETE or 8(S),15(S)-diHpETE, respectively [26]. This model is not applicable to the oxidation of ALA and ARA catalyzed by PhLOX, because the two fatty acids both produce 9,12-dioxo products despite their different carbon chain lengths. However, if the reactants can enter the substrate-binding pocket from different directions (ALA from the carboxyl end first and ARA from the methyl end first), the oxygen insertion points will match the respective final product. For instance, the carboxyl end of a C18 fatty acid and the methyl end of a C20 fatty acid entered into the pocket. The catalytic reaction was performed in two steps. In the first step, a hydrogen atom was abstracted from the C-11 carbon atom of a C18:3 fatty acid, with subsequent introduction of oxygen in the n-2 position to generate 9(S)-HpOTE. For ARA, a hydrogen atom is abstracted from the ω-11 carbon atom and then molecular oxygen inserted into the C-12 position to form 12(S)-HpETE. After formation of the first step product, the catalytic target group was moved forward by three carbons (C14 in C18:3 fatty acid or ω-C14 in C20:4 fatty acid) through a conformational shift by the orientation of Phe826 and Phe642. In the second step, a hydrogen atom was abstracted from the C-14 carbon atom of 9(S)-HpOTE and, subsequently, an oxygen inserted into the C-12 position to form 9,12-diHpOTE. For a C20:4 fatty acid, a hydrogen atom was abstracted from the ω-14 carbon atom and then molecular oxygen inserted into the ω-12 position to form 9,12-diHpETE.

AOS is a hemoprotein that belongs to the CYP74 family [17], whereas LOXs are nonheme iron-containing dioxygenases [19]. LOX-AOS fusion proteins found in cyanobacteria and corals have additional AOS domains outside of their LOX active pockets [15,16]. However, the 3D model of PhLOX showed only one LOX active pocket in this multifunctional enzyme. Thus, it can be inferred that the AOS function of PhLOX may have a similar but different substrate conversion mode to that of the LOX-AOS enzyme in catalyzing fatty acids or hydroxylated fatty acids into ketol products. In the AOS of *Arabidopsis thaliana* (AtAOS), the peroxy group of the substrate ligates the heme and interacts with the catalytic Asn321, and the strictly conserved Phe137 interacts with the incipient carbocation through cation–π interactions to stabilize the carbon-centered radical [17]. In the oxidation of ARA catalyzed by PhLOX, the amide group of Gln777 is only ~2.4 Å away from the epoxide oxygen in 8(S),9(R)-EET, and the AOS function of PhLOX was eliminated when Gln777 was replaced by leucine. In addition, no phenylalanine exists around the catalytic sites of PhLOX, although the nearby imidazole ring of His583, which can bind ferric iron, may engage in cation–π interactions as its π ring is only ~4 Å away from the C-10 position of 8(S),9(R)-EET. However, Gln777 and His583 are both conserved in animal and plant LOXs, and they cannot explain why the AOS function is unique to PhLOX. Among the unique amino acids in red algae LOX, Asn575 has its amide nitrogen close to the carboxylic end of fatty acid substrates, and the AOS activity of PhLOX diminished when leucine as a neutral residue replaced Asn575. It is likely that some uniquely evolved amino acids (such as Asn575) are combined with conserved amino acids (such as Gln777 and His583) for PhLOX to realize AOS-like function in one active pocket.

LOXs from higher eukaryotes have an additional domain at their N-terminus, referred to as the PLAT (Polycystin-1, Lipoxygenase, Alpha-Toxin) domain, that is implicated to bind to small molecule allosteric effectors to in turn modulate substrate specificity and the rate-limiting steps of catalysis [27]. By analyzing the evolution of the functional domain of LOXs, Chen et al. reported that the N-terminal SRPBCC domain, instead of the PLAT domain, was present in some LOXs from Rhodophyta and marine bacteria [10] and speculated that the SRPBCC domain and the LOX part of *Pyropia* may have been acquired from marine bacteria by horizontal gene transfer [8,10]. For SvLOX and some Bangiales LOXs, the SRPBCC domain is conserved at the N-terminal, but such conservation is not observed in Florideophyceae LOXs such as CcLOX, and the enzyme activity likely changes to some extent as a result. Many observations suggested that the SRPBCC domain is key to the HPL function. For example, both PhLOX and SvLOX have the SRPBCC domain, and they both have HPL activity. In contrast, CcLOX lacks the SRPBCC domain and does not give HPL products. In addition, when only the first beta barrel structure of the SRPBCC domain was deleted (PhLOX-C0), the enzyme exhibited stronger ability to produce C8-VOC from ARA, but it lost its HPL activity completely when the SRPBCC domain was deleted in its entirety (PhLOX-C1). That is, the second beta barrel of the SRPBCC domain may have some impact on the substrate channel or the protein space of the C-terminal functional domain. Garreta et al. reported that in soybean 9-LOX, rabbit 15-LOX, and *P. aeruginosa* 10-LOX, a mobile channel for the entry of oxygen exists on the opposite side of the nonheme iron [28]. This channel connects to the region with high oxygen affinity active sites on the protein surface and regulates the specificity of reactions. When the SRPBCC domain is cut off in the 3D model of PhLOX, the helices in the connecting area will deflect. In particular, helix5 and helix6, which are both connecting units in the middle, will swap position with the active pocket of PhLOX after the loss of the SRPBCC domain. This kind of spatial movement likely alters the entry of the substrate or oxygen into the active pocket of the engineered enzymes.

However, PhLOX2 does not have HPL activity, even though it is on the same evolutionary clade with PhLOX and retains the SRPBCC domain on its N-terminal [11]. Instead, PhLOX2 has hydroperoxidase activity like SvLOX and CcLOX. This functional discrepancy may be related to the differentiation of certain amino acids and their role in orienting substrates. The production of volatiles from ARA was increased when Leu replaced Asn575, Gln777, or Phe826 in PhLOX. That is, the removal of some positioning factors in PhLOX reoriented the substrate to favor the HPL activity. Alignment shows that Asn575, Gln777, and Phe826 are conserved among PhLOX, PhLOX2, and CcLOX, and the amino acid identity is 50.33% between PhLOX and PhLOX2 and 33.33% between PhLOX and CcLOX. Therefore, substrates are probably oriented very differently in the active pocket of PhLOX2 compared to in PhLOX, which may be why neither PhLOX2 nor CcLOX has HPL activity. During the expansion of the Porphyra LOX gene family, although the invariable SRPBCC domain provided some functional inheritance, some amino acids were likely differentiated to alter the HPL activity. In fact, LOXs with hydroperoxidase activity are also common in lower organisms such as cyanobacteria and fungi. For example, the cyanobacteria *Acaryochloris marina* and *Anabaena* sp. have LOXs that can catalyze the oxidation of ALA, giving 9-HpOTE and 9-hydroxy octadecatrienoic acid (9-HOTE) after oxygen insertion at the C-9 position [29,30], and the oxidation of ARA is catalyzed by the 15-LOX of the fungus *Saprolegnia parasitica* to give 15-HPETE and 15-HETE [22]. Since the catalytic reactions of PhLOX2 and CcLOX give hydroxylation products, their functional evolution mainly retains the hydroperoxidase activity, which the LOXs of the ancestors of marine Proteobacteria have in common, but the multifunctional evolution of PhLOX is related to HPL instead. In addition, no ketol products existed in any of the catalytic reactions of SvLOX and CcLOX, which ruled out the AOS function. Their lack of the AOS function is likely related to the positioning effect of certain amino acids. For example, when aligned to PhLOX by Arg792 and Phe826, SvLOX has neutral amino acids at both positions (Ala739 and Leu770) and CcLOX has Phe for both.

In conclusion, Asn575, Phe642, Gln777, and Phe826 in PhLOX jointly regulated substrate orientation in the active pocket, to realize the double peroxidation of fatty acids and, when the AOS function is in effect, the conversion of hydroperoxides to ketols. The HPL function of PhLOX is inherited from a marine Proteobacteria ancestor, and PhLOX regulates VOC production by modifying its N-terminal SRPBCC domain. With the combinations of functional domains and amino acids, some LOXs of the *Pyropia*/*Porphyra* family developed impressive multifunctionality. This work provides new insights into the evolution of the oxylipin metabolism in algae and how LOXs help them adapt to complex intertidal environments.

## 4. Materials and Methods

### 4.1. Structural Modeling

The tertiary protein structure of PhLOX was predicted in silico using Robetta (https://robetta.bakerlab.org/) (accessed on 16 November 2023) and SWISS-MODEL (https://swissmodel.expasy.org/) (accessed on 26 November 2020). The crystal structure of soybean LOX-3 was aligned to the 3D model of PhLOX to extract the Fe metal and 13(S)-HpODE ligand. Based on the extracted 13(S)-HpODE, the molecular configuration was calculated analogously for 9(S)-HpOTE, 12(S)-HpETE, and 8(S),9(R)-EET. These three ligands were docked into PhLOX using Gold 5.141 (Cambridge Crystallographic Data Centre, Cambridge, UK). All amino acid residues within 10 Å to the ligand were selected as active sites. The default settings were used for other parameters.

### 4.2. Multiple Sequence Alignment and Phylogenetic Analysis

The amino acid sequences of LOXs from prokaryotes, algae, higher plants, and mammals were retrieved from the NCBI database and multi-aligned using the MUSCLE method in MEGA 7 (Mega Limited, Auckland, New Zealand). Evolutionary analysis was then conducted using the neighbor joining method, and the matrix of pairwise distances was built based on the Poisson model with a bootstrap of 1000 replicates.

### 4.3. Site-Directed Mutagenesis

Several specially designed amplification primers were used to obtain site-directed mutant sequences of PhLOX at the sites Asn575, Phe642, Gln777, and Phe826 (Table S2). The upstream and downstream paired overlapping mutant sequences were amplified using the recombinant pET28a-*Phlox* plasmid [10] as the template. The AccuPrime™ Pfx polymerase (Invitrogen, Carlsbad, CA, USA) was used for PCR, and the procedure included denaturation at 95 °C for 3 min, annealing for 28 cycles at 95 °C for 30 s, 58 °C for 30 s, and 68 °C for 90 s, and final extension at 68 °C for 10 min. The obtained pairs of PCR fragments were purified and used as the template in splicing using the primers L1-1117F/L1-2697R and the following procedure: 95 °C for 3 min, 28 cycles of 95 °C for 30 s, 58 °C for 30 s, and 68 °C for 2 min, and finally 68 °C for 10 min. The splicing products were purified and sequenced. To obtain the F642V-F826L mutant, the F826L mutant sequence was used as the template. The corresponding upstream and downstream mutant sequences were amplified with F-F642V-1909F/L1-2697R and L1-1117F/F-F642L-1941R, respectively, and the splicing experiment was carried out similarly afterwards.

### 4.4. Sequence Cloning and Synthesis

The specific primers were designed (Table S2) and PrimeSTAR^®^ Max DNA Polymerase (Takara, China) was used to amplify four *Phlox* sequences of different size that were shortened at the 5′-terminal (i.e., *Phlox-C0*, *Phlox-C1*, *Phlox-C2*, *Phlox-C3*). The recombinant pET28a-*Phlox* plasmid was used as the template, and the PCR procedure included 30 annealing cycles of 98 °C for 10 s, 62.5 °C for 5 s, and 72 °C for 10 s. The PCR products were purified and sequenced. The coding sequences of *S. violacea lox* (gene ID BAJ02412) and *C. crispus lox* (gene ID CDF77589) were synthesized and recombined into the cloning vector pUC57 at Sangon Biotech (Shanghai, China) Co., Ltd. to obtain pUC57-*Svlox* and pUC57-*Cclox*.

### 4.5. Prokaryotic Expression and Purification

All site-directed mutant PCR products were digested and ligated onto the corresponding PstI/HindIII-digested pET28a-*Phlox* plasmid, and then transformed into *E. coli* BL21 cells to express a recombined protein carrying an N-terminal His-tag. In addition, the four 5′-terminal shortened sequences, the pUC57-*Svlox* plasmid, and the pUC57-*Cclox* plasmid were digested with NdeI/HindIII, BamHI/NotI, and NdeI/XhoI, respectively, and the obtained materials were ligated onto the corresponding pET28a vector (after restriction enzyme digestion) and transformed into *E. coli* BL21 cells to obtain the recombinant cells. Next, the transformants were induced in the presence of 0.1 mM isopropyl thio-β-galactoside (Sigma-Aldrich, Shanghai, China), and the resulting cells were harvested and lysed. After centrifugation at 13,000 rpm for 15 min, the supernatant was purified using a Ni-agarose column (His-Tagged Protein Purification Kit, CWBIO, Beijing, China) to collect the recombinant enzymes.

### 4.6. Enzymatic Oxidation of PUFAs

Linoleic acid (LA), ALA, ARA, ARA-[D8], and EPA were purchased from Cayman Chemical (Ann Arbor, MI, USA). The fatty acid (100 μM) was added to the solution of the pure enzyme (0.1 mg·mL^−1^, 1 mL) at 20 °C. For HPLC-MS sample preparation, the mixture was extracted immediately with 1 mL ethyl acetate after incubation for the set time. The extract was shaken at 1100 rpm and 4 °C for 20 min, and centrifuged at 12,000 rpm at 4 °C for 10 min. The upper layer was collected and the lower water phase was extracted again. Organic phases from two collections were combined and dried with a stream of nitrogen, redissolved in methanol (500 µL), and analyzed by HPLC-MS.

Alternatively, the reaction was terminated at defined time points by adjusting pH < 2 with 6 M HCl, and volatile organic compounds (VOCs) were gathered from the mixture at 40 °C for 50 min using a divinylbenzene/carboxen/polydimethylsiloxane solid phase microextraction (SPME) device (Supelco Inc., Stockbridge, GA, USA) for GC-MS detection.

### 4.7. Catalysis on Hydroperoxy Fatty Acids

9(S)-HpOTE and 12(S)-HpETE were purchased from Cayman Chemical (Ann Arbor, MI, USA). The hydroperoxy fatty acid (50 μM) was used as the substrate of PhLOX (1 mL, 0.1 mg·mL^−1^) at 20 °C for 15 min. Then, the reaction was terminated and prepared for HPLC-MS or GC-MS, followed as above.

### 4.8. HPLC-MS/MS Analysis

Samples were analyzed on an UltiMate 3000 Basic HPLC System (Thermo Fisher, Waltham, MA, USA) interfaced with a Q Exactive hybrid quadrupole–Orbitrap mass spectrometer using a Hypersil GOLD C18 column (2.1 mm × 100 mm, 3 μm, Thermo Fisher, Waltham, MA, USA). The HPLC conditions were as follows: injection, 1 μL; column temperature, 30 °C; gradient elution with (A) acetonitrile and (B) 0.1% formic acid from 30% A to 70% A over 40 min; flow rate, 0.1 mL·min^−1^. The data-dependent mode was operated to automatically switch between full scan MS and MS/MS acquisition to perform high-resolution mass spectrometry. The MS conditions were as follows: electrospray ionization (ESI), negative ion mode; full scan MS spectrum, *m*/*z* = 100–500; resolution, 70,000 (m/z 200); AGC target, 1 × 10^6^; dynamic exclusion, 70 s; maximum ion time, 250 ms; spray voltage, 2 kV; no sheath or auxiliary gas; heated capillary temperature, 275 °C. The MS/MS parameters were set as follows: AGC target 2 × 10^5^; maximum ion time 250 ms; isolation width 2 Da. The α/γketols were detected in SIM (single ion monitoring) by monitoring *m*/*z* = 311.22, 309.20, 335.22, or 333.20 for corresponding products derived from LA, ALA, ARA, or EPA, respectively.

Commercial hydroxy and hydroperoxy fatty acids, including 9(S)-hydroperoxy-10(E),12(Z)-octadecadienoic acid (i.e., 9(S)-HpODE), 13(S)-HpODE, 9(S)-HpOTE, 13(S)-hydroperoxy-9(Z),11(E),15(Z)-octadecatrienoic acid (i.e., 13(S)-HpOTE), 8(S)-HETE, 12-HpETE, 8(S)-HEPE, 12-hydroperoxy eicosapentaenoic acid (12-HpEPE), were purchased from Cayman Chemical (Ann Arbor, MI, USA), formulated at a concentration of 25 μM in 0.5 mL methanol, analyzed by HPLC-MS as described above, and then used as standard compounds for the comparison of MS/MS spectra of analytes (Table S3).

### 4.9. GC-MS Analysis

The volatile components of PhLOX-catalyzed samples were analyzed using a reported Headspace SPME/GC-MS method [31], with slight modifications. Samples were analyzed on an 8890-5977B GC-MS system (Agilent, Santa Clara, CA, USA) using an HP-5MS UI column (30 m × 0.25 mm, 0.25 μm, Agilent, Santa Clara, CA, USA). The SPME device used to extract the VOCs was introduced in a GC splitless injector and maintained at 280 °C for 5 min. The carrier gas was helium, and the flow rate was 1 mL·min^−1^. The temperature gradient was programmed as follows: 35 °C for 5 min, increased to 50 °C at 3 °C min^−1^, 50 °C for 2 min, increased to 180 °C at 6 °C min^−1^, 180 °C for 2 min, increased to 280 °C at 20 °C min^−1^, and finally 280 °C for 5 min. Mass spectra (*m*/*z* = 50–500) were obtained using electron ionization (70 eV). The ion source temperature was 230 °C, and the interface temperature was 280 °C.

Commercial volatile aroma compounds (1-octen-3-ol, 1-octen-3-one, 2*E*-nonenal and 2*E*,4*E*-decadienal) were purchased from Sigma-Aldrich (Merck KGaA, Darmstadt, Germany) and used as standard compounds. They were mixed with concentrations ranging from 1 to 100 μM, and then handled by the same procedure of SPME and GC-MS described above to construct the standard curve. Both retention time and mass spectra of analytes were compared with those of the standard compounds. The mass spectra of analytes were also compared with the standard spectra recorded in the NIST 147 and the WILEY 7 Spectrometry Libraries. The contents of volatile products were calculated from the GC peak areas by using the standard curve method.

### 4.10. Time Course of Enzyme Activity

The fatty acid (100 μM) was added to the solution of the pure enzyme (0.1 mg·mL^−1^, 200 µL) in a 96-well microplate at 20 °C. The enzyme activity was then immediately determined by monitoring the UV absorbance of hydroxy and hydroperoxy products at 235 nm (conjugated diene system of the hydroxy fatty acids) with a VarioskanLUX microplate reader (Thermo Fisher, USA). The absorbance at 235 nm was read automatically every 10 s, and the default settings were used for other parameters.

## Figures and Tables

**Table 1:** Yield of volatile products after incubation arachidonic acid with PhLOX and its mutants (μM).

Products	By PhLOX	By Mutants			
		N575L	Q777L	F826L	F624V-F826L
1-octen-3-ol	19.5 ± 2.9	45.4 ± 5.2	42.9 ± 3.2	67.1 ± 5.2	ND ^1^
1-octen-3-one	18.3 ± 3.8	36.0 ± 3.1	18.4 ± 1.1	21.2 ± 2.4	ND
2-nonenal	16.0 ± 1.5	10.0 ± 0.7	18.2 ± 1.3	29.5 ± 3.3	ND

^1^ Not detected.

## Data Availability

The original contributions presented in this study are included in the article/Supplementary Materials; further inquiries can be directed to the corresponding authors.

## Supplementary Materials

The following supporting information can be downloaded at https://www.mdpi.com/article/10.3390/ijms252010956/s1.
