# Supplementary material for: Intricate Evolution of Multifunctional Lipoxygenase in Red Algae
Source: Int J Mol Sci. 2024 Oct 11;25(20):10956. doi: 10.3390/ijms252010956 (PMC11507377; doi:10.3390/ijms252010956)

Article

Intricate evolution of multifunctional lipoxygenase in red algae

Zhujun Zhu [1,2], Yanrong Li [1], Xinru Wu [2], Jia Li [2], Xiaodong Mo [2], Xiaojun Yan [3], and Haimin Chen [2,*]

[1] Marine Drugs and Biological Products Department, Ningbo Institute of Oceanography, Ningbo 315832, China; zhuzj@nbio.org.cn
[2] State Key Laboratory for Managing Biotic and Chemical Threats to the Quality and Safety of Agro-products, Ningbo University, Ningbo 315832, China; chenhaimin@nbu.edu.cn
[3] Collaborative Innovation Center for Zhejiang Marine High-efficiency and Healthy Aquaculture, Ningbo University, Ningbo 315832, China; yanxiaojun@nbu.edu.cn
* Correspondence: chenhaimin@nbu.edu.cn; Tel.: +85 0574 89281049

Supplementary Material

Supplementary Figures

Figure S1. Conserved domains of PhLOX, SvLOX, and CcLOX based on the NCBI CDD database.

Figure S2. Product specificity of PhLOX on hydroperoxide intermediates.

Figure S3. Ketol products formed from the reaction of PhLOX with different fatty acids and their identified fragment ions.

Figure S4. GC-MS analysis of short chain volatiles produced after 12(S)-HpETE catalysis by PhLOX.

Figure S5. GC-MS detection of volatile products from the oxidation of ARA.

Figure S6. 3D models of PhLOX of different size derived with Robetta.

Figure S7. Transcript isoforms of PhLOX.

Supplementary Tables

Table S1. Major molecular ions identified for different fatty acid derives after enzyme catalysis ($(M\text{-}H)^-$).

Table S2. Primers for the cloning of site-directed mutants of PhLOX and its N-terminal shortened isoforms.

Table S3. Major molecular ions of hydroxy and hydroperoxy fatty acids for which standards are commercially available ($[M\text{-}H]^-$).

Figure S1. Conserved domains of PhLOX, SvLOX, and CcLOX based on the NCBI CDD database.

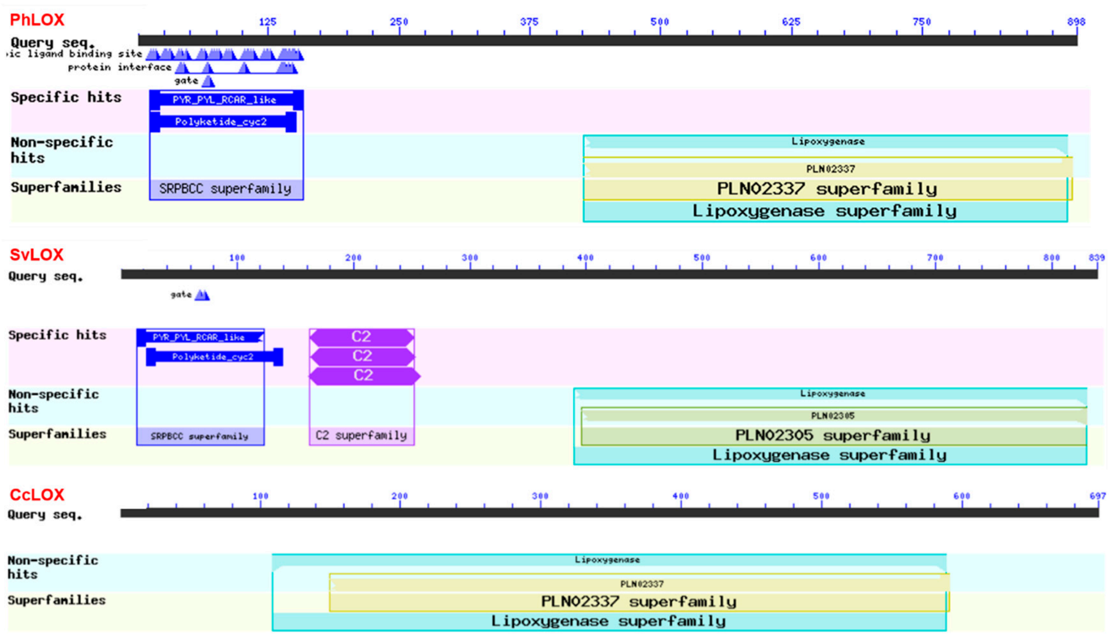

Figure S2. Product specificity of PhLOX on hydroperoxide intermediates. (a) 9(*S*)-hydroperoxy octadecatrienoic acid (9(*S*)-HpOTE) as substrate incubated in 1 mL 0.1 mg mL^{-1} of PhLOX (the wild type, WT) at 20°C for 15 min. (b) and (c) Negative ionization mass spectra for products by HPLC-MS/MS.

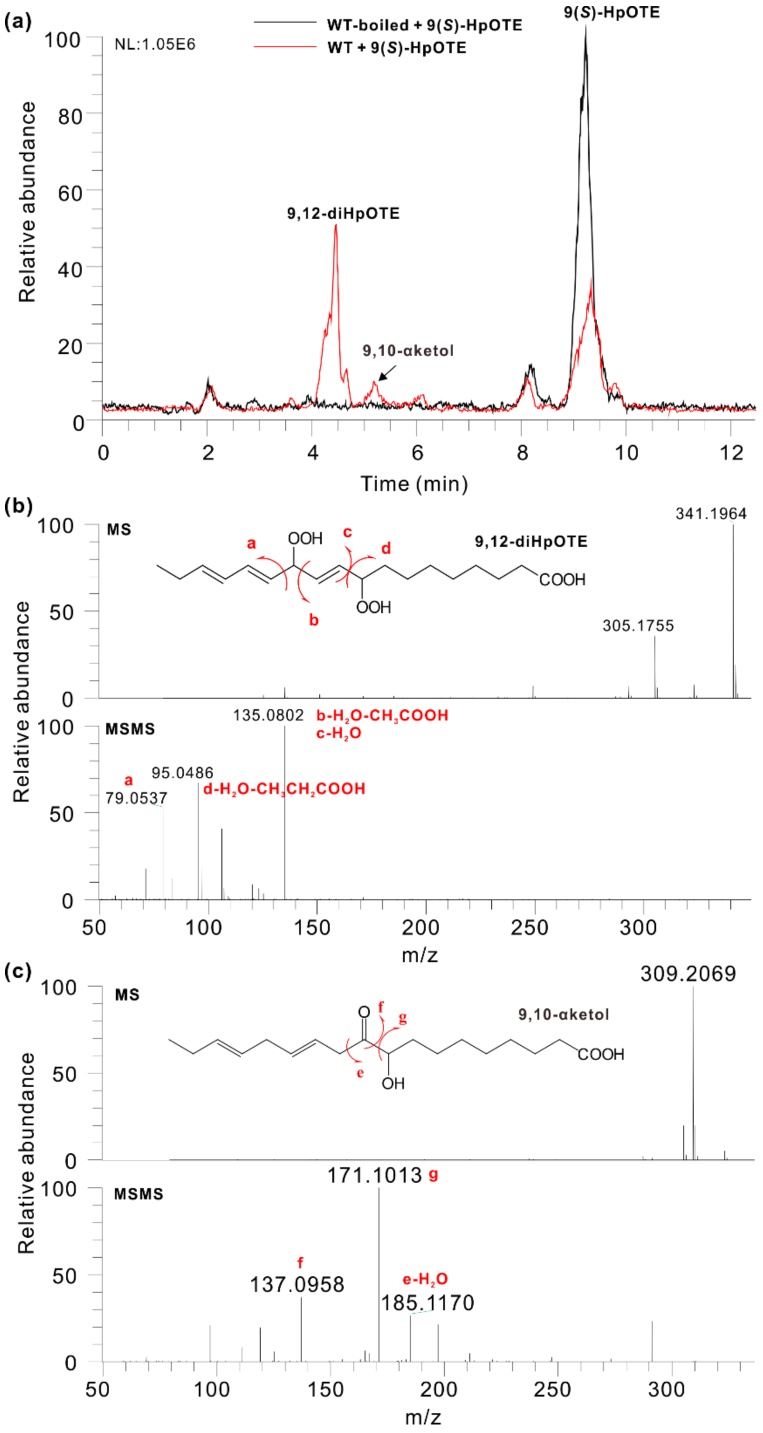

Figure S3. Ketol products formed from the reaction of PhLOX with different fatty acids and their identified fragment ions. (a) Single ion monitoring (SIM) was used to detect ketol products produced by PhLOX, using different fatty acid (100 µM) as substrates at pH 8.0 and 20 °C for 15 min. (b) Negative ionization mass spectra for ketol products by HPLC-MS/MS. Furthermore, isotopically-labeled [D8]-arachidonic acid (ARA-[D8]) was used as substrate for PhLOX and the corresponding ketol was identified.

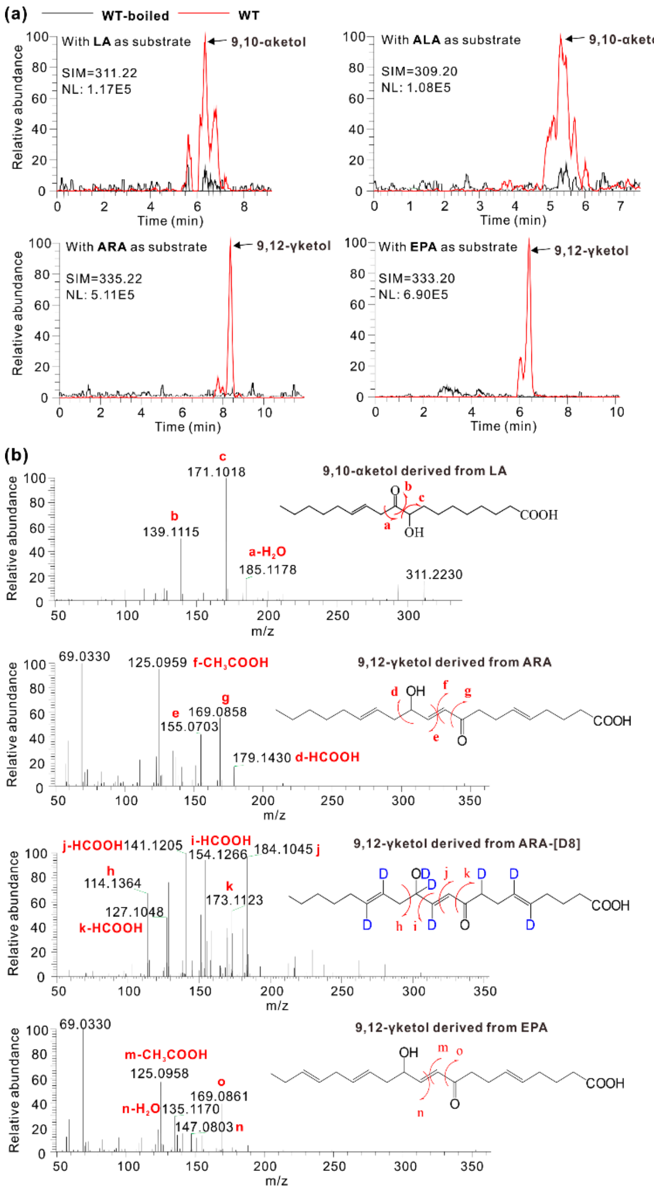

Figure S4. GC-MS analysis of short chain volatiles produced after 12(S)-HpETE catalysis by PhLOX.

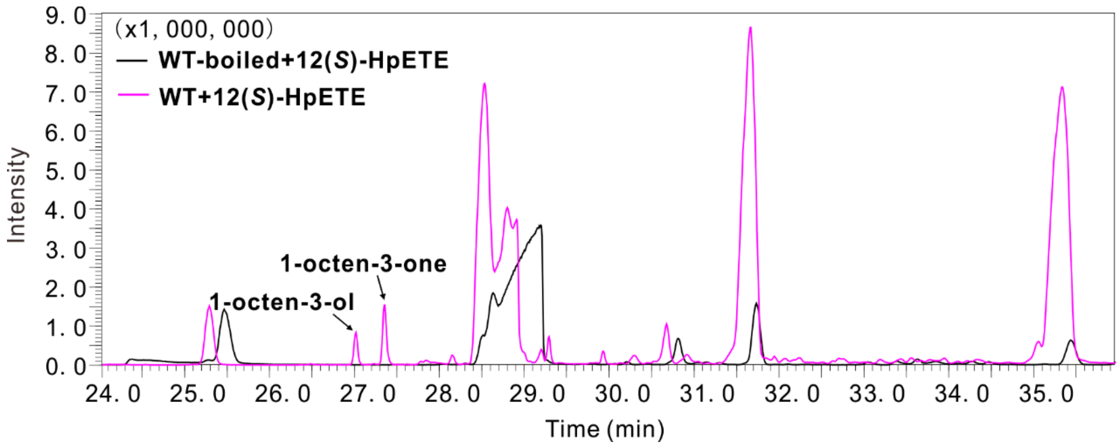

Figure S5. GC-MS detection of volatile products from the oxidation of ARA. ARA (100 μM) was incubated with the enzyme (0.1 mg mL^{-1}) at pH 8.0 and 20 °C for 15 min. The enzymes included wild-type (WT) PhLOX and the N575L, Q777L, F826L, and F642V-F826L mutants.

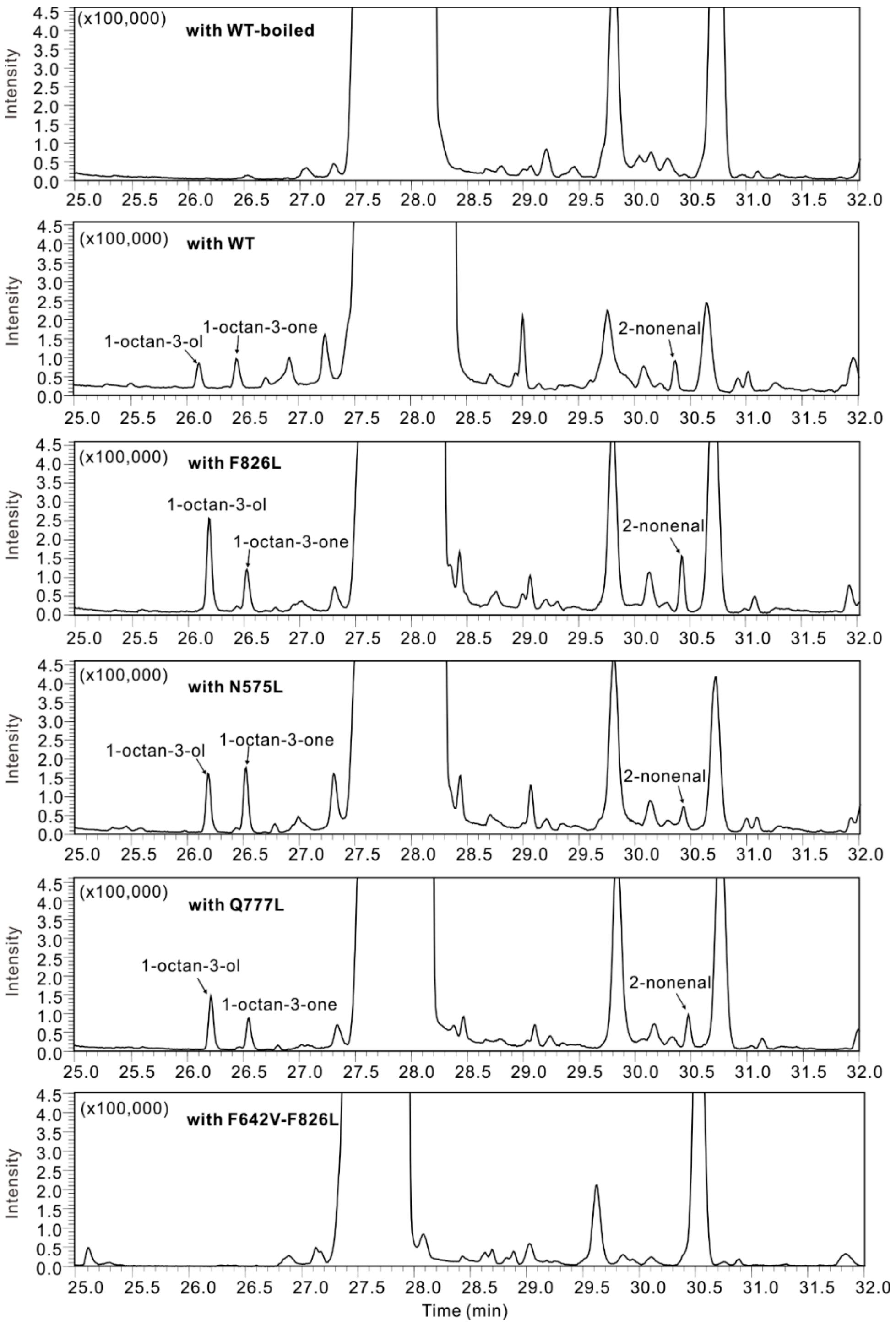

Figure S6. 3D models of different PhLOX without the N-terminal SRPBCC domain derived with Robetta

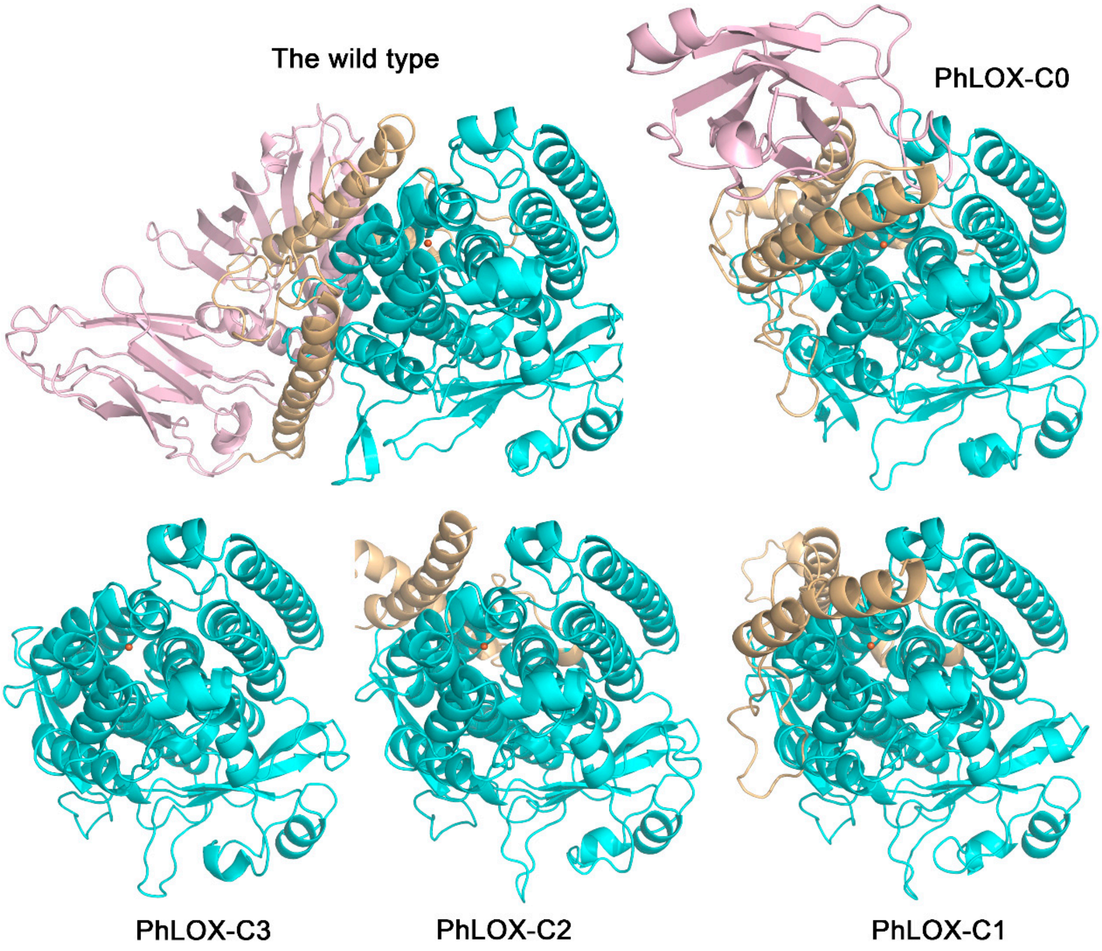

Figure S7. Transcript isoforms of PhLOX. The sequence range related to non-coding region (UTR), coding sequence (CDS), lipoxygenase superfamily functional domain, and SRPBCC domain is colored in green, yellow, pink, and blue, respectively.

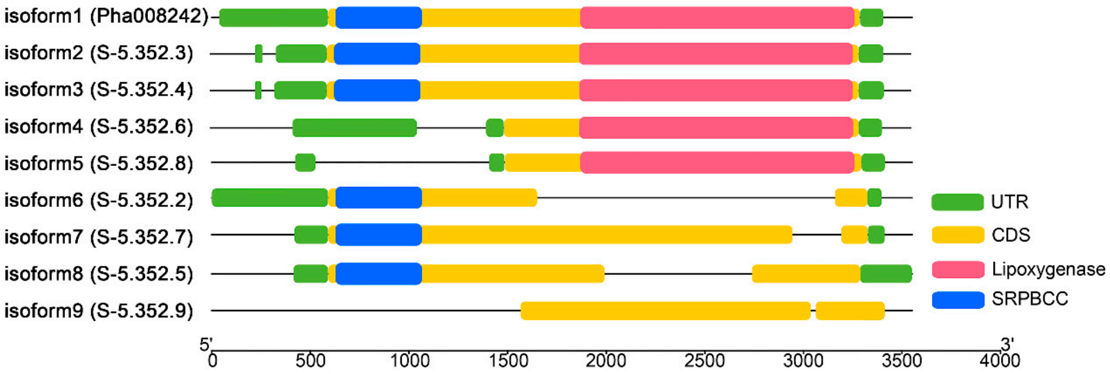

Supplement: Supplementary file 1 [file ijms-25-10956-s001.zip › Supplementary figures.pdf]
